# A systematic review on the prospects of X- and Y-sexed semen in ruminant livestock: implications for conservation, a South African perspective

**DOI:** 10.3389/fvets.2024.1384768

**Published:** 2024-04-09

**Authors:** Jabulani Nkululeko Ngcobo, Tshimangadzo Lucky Nedambale, Sindisiwe Mbali Sithole, Bohani Mtileni, Takalani Judas Mpofu, Fhulufhelo Vincent Ramukhithi, Tlou Caswel Chokoe, Khathutshelo Agree Nephawe

**Affiliations:** ^1^Department of Animal Sciences, Tshwane University of Technology, Pretoria, South Africa; ^2^Germplasm, Conservation, Reproductive Biotechnologies, Agricultural Research Council, Pretoria, South Africa; ^3^Department of Agriculture, Land Reform, and Rural Development, Directorate Farm Animal Genetic Resource, Pretoria, South Africa

**Keywords:** pre-gender selection, indigenous ecotypes, fertility, flow cytometry, artificial insemination, conservation, improving sexed semen

## Abstract

South Africa is home to numerous indigenous and locally developed sheep (Nguni Pedi, Zulu, and Namaqua Afrikaner, Afrino, Africander, Bezuidenhout Africander, Damara, Dorper, Döhne Merino, Meat Master, South African Merino, South African Mutton Merino, Van Rooy, and Dorper), goat (SA veld, Tankwa, Imbuzi, Bantu, Boer, and Savanna) and cattle (Afrigus, Afrikaner, Bolowana, Bonsmara, Bovelder, Drakensberger, South African Angus, South African Dairy Swiss, South African Friesland, South African Red, and Veld Master) animals. These breeds require less veterinary service, feed, management efforts, provide income to rural and or poor owners. However, most of them are under extinction risks and some with unknown status hence, require immediate conservation intervention. To allow faster genetic progress on the endangered animals, it is important to generate productive animals while reducing wastages and this can be achieved through sex-sorted semen. Therefore, this systematic review is aimed to evaluate the prospects of X and Y-sexed semen in ruminant livestock and some solutions that can be used to address poor sex-sorted semen and its fertility. This review was incorporated through gathering and assessing relevant articles and through the data from the DAD-IS database. The keywords that were used to search articles online were pre-gender selection, indigenous ecotypes, fertility, flow cytometry, artificial insemination, conservation, and improving sexed semen. Following a careful review of all articles, PRISMA guidelines were used to find the articles that are suitable to address the aim of this review. Sex-sorted semen is a recently introduced technology gaining more attention from researchers particularly, in the conservation programs. Preselection of semen based on the sex chromosomes (X- and or Y-bearing chromosomes) is of paramount importance to obtain desired sex of the offspring and avoid animal wastage as much as possible. However, diverse factors can affect quality of semen of different animal species especially after sex-sorting. Flow cytometry is a common method used to select male and female sperm cells and discard dead and abnormal sperm cells during the process. Thus, sperm sexing is a good advanced reproductive technology (ART) however, it is associated with the production of oxidative stress (OS) and DNA fragmentation (SDF). These findings, therefore, necessitates more innovation studies to come up with a sexing technology that will protect sperm cell injuries during sorting in frozen-thawed.

## 1 Introduction

South African households are mostly food insecure with ±20% considered as below bread level (1). Climate change on the other hand cannot be underestimated due to its immense negative impact on the global south countries (2). Indigenous and locally developed ruminants play a vital role in commercial and subsistence farming levels (3). In commercial set-up farming, indigenous and locally developed ecotypes are used for meat and hide production, sales, and export whereas at the subsistence level, they are used as a source of meat, cash, milk and to pay lobola and also used to pay penalties to rural authorities (4).

The South African Development Community (SADC) region possesses ~38 million goat populations that are kept by resource-limited farmers (5). Goats are kept by rural farmers mainly as a source of income through live sales, meat, and hide production (6). Akinmoladun et al. (7) found that goats can quickly adapt to water stress and do not lose body weight easily, which extends their survival. On the other hand, sheep are kept as a source of protein, of income, to pay lobola, and used for traditional ceremonies (8). Cattle are also used to pay lobola and to pay penalties to the rural authorities. However, the Domestic Animal Diversity Information System (DAD-IS) (9) shows that most of the indigenous South African livestock ruminants are endangered with their population declining daily, necessitating urgent interventions through advanced reproductive biotechnologies (ART).

There are recently introduced ARTs to conserve endangered species such as estrous synchronization, artificial insemination, and embryo transfer (10). The pre-selection of the sex plays an important role in increasing genetic gain and the selection of the desired sex (11). The sex-sorting concept has sparked a rising interest in calf sex pre-selection using sexed semen in both dairy and beef farmers worldwide where artificial insemination (AI) is used (12). This process is qualified due to the high demand for heifer calves by the dairy industry to be used as replacement heifers. However, there are considerable limitations to getting complete and accurate information on how many doses of sexed semen are given yearly to AI cows (13), the method of sex sorting, and the challenges associated with sexing semen.

There are numerous developed methods used to sex semen for livestock ruminants, including albumin gradient/gradient swim down procedure, the Percoll density gradient method, swim up procedure, free flow electrophoresis, the identification of H–Y antigen, sperm sorting based on the volumetric differences, centrifugal counter current distribution, immunological approaches, proteomics approaches, and flow cytometry (14). Flow cytometry is the only commercialized method of sexing semen and uses LASER to stimulate fluorescent dye that fixes the DNA in spermatozoa. Magopa et al. (12) reported an overall conception rate of 61% and 62% when X-sexed and unsexed sperm were used, respectively, in dairy cows in comparison to 56.0% and 52.2% in beef cows. However, there were embryo losses between days 35 and 65 in dairy cows (X-sexed, 33.3% and unsexed, 18.2%) and beef cows (X-sexed, 28.6% and un-sexed, 29.2%).

In sheep and goats, artificial insemination with sex-sorted semen is still scarce and is only practiced in biotechnology companies. It can be assumed that the less use of sex-sorted semen in goats is due to low fertility in goats following the use of artificial insemination and not exceeding 50% (15). These challenges (poor fertility and poor sperm quality) are factors that continue to raise concerns or arguments about the outcomes or relevance of sex sorting techniques in sheep and goats entailing the need to further address and ensure that the sex sorting technology is understood. The Y-chromosome-bearing sperm cell is smaller in size, has less DNA, high motility, and less density (16). This is the type of sperm cell required mostly in feedlot setup where males are targeted for their higher growth rate and body size (17). Therefore, this systematic review is aimed to evaluate the prospects of X- and Y-sexed semen in ruminant livestock and provide some solutions that can be used to address poor sex-sorted semen and its fertility.

## 2 Methodology

**Figure 1:**
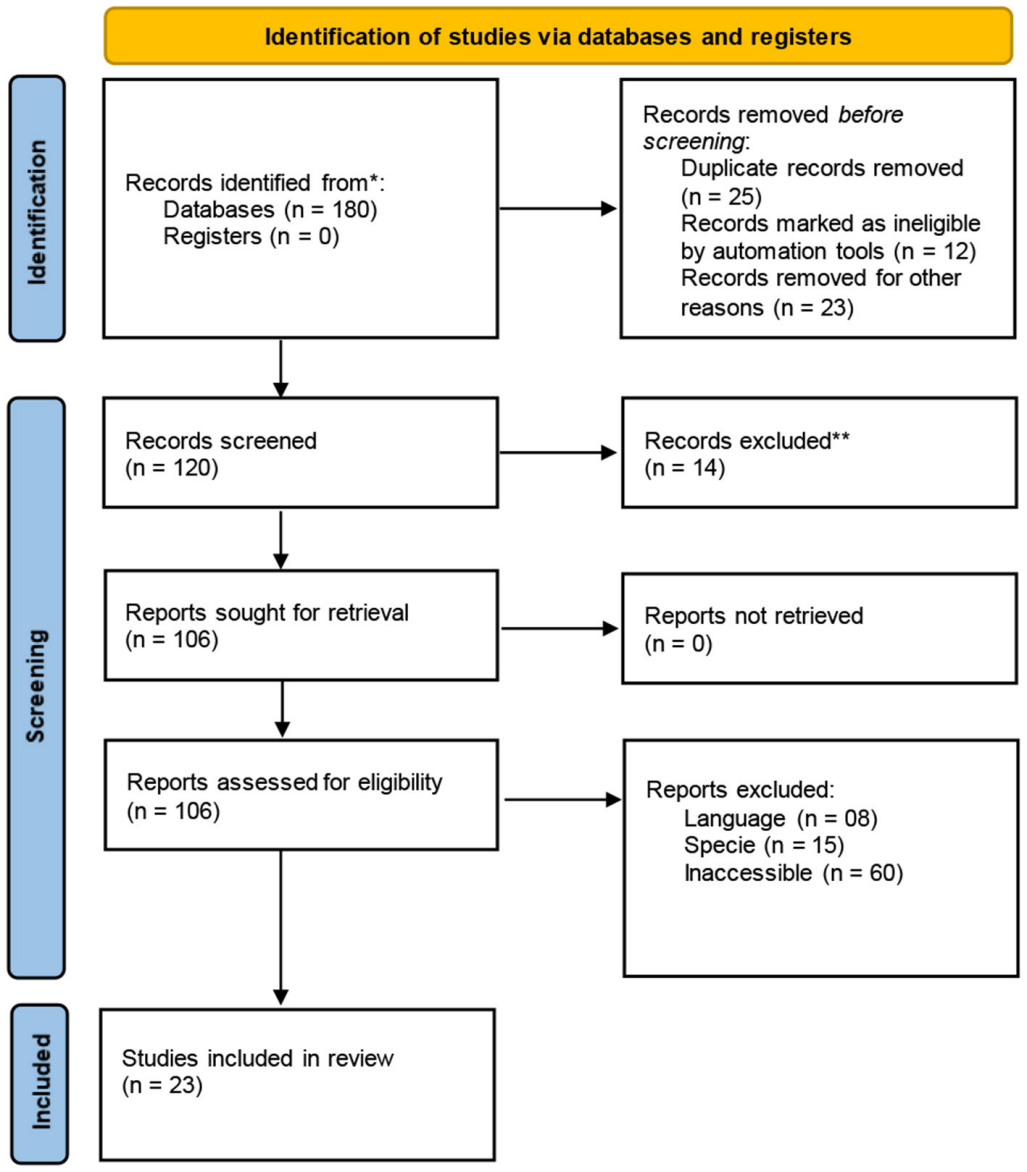
PRISMA flow diagram (18).

The purpose of this systematic review was to review the prospects of X- and Y-sexed semen in ruminant livestock and its possible role in saving engendered ruminants. Relevant articles were gathered and assessed, and the data from the DAD-IS database was used in this review. The keywords that were used to search articles online were pre-gender selection, indigenous ecotypes, fertility, flow cytometry, artificial insemination, conservation, and improving sexed semen. Following a careful review of all articles to find suitable articles, PRISMA guidelines were used as described by Haddaway et al. (18) (see Figure 1).

## 3 Literature search

To gather all articles assessing sexed or sex-sorted semen in cattle, sheep, and goats, an online database search was conducted. One of the search combinations was used:

1. Pre-gender selection (sheep or cattle or goats)
2. Methods of sexing semen (flow cytometry, sex-sorted semen in sheep, cattle, and goats).
3. Improving sex-sorted/sexed semen [sexed-sex-sorted semen and antioxidants (vitamin C or ascorbic acid)].
4. Sex-sorted semen (conservation of cattle, sheep, and goats).
5. Pregnancy (accuracy of sexed or sex-sorted semen in cattle, sheep, and goats).
6. Sexed or sex-sorted semen preservations (liquid preservation or frozen-thawed semen).
7. Sexed or sex-sorted semen (challenges in cattle, sheep, and goats).
8. Pregnancy loss following sexed or sex-sorted semen (cattle, sheep, and goats).
9. Sexed or sex-sorted semen antioxidants capacity (superoxide dismutase, catalase, glutathione peroxidase, and glutathione reductase in sheep, cattle, and goats).
10. Sexed or sex-sorted semen proteomes (shift in cattle, sheep, and goats sexed or sex-sorted semen).
11. Improving sexed or sex-sorted semen (antioxidants in cattle, sheep, and goats).
12. Nanotechnology and sexed or sex-sorted semen (cattle, sheep, and goats).

These terms were entered in PubMed, Google Scholar, Scopus, VetMed resources, and ScienceDirect databases. In Google Scholar, the search was made to retract articles published between 2000 and 2024. In all these databases, the search was only limited to those articles written in English, while those in other languages, e.g., Spanish, Portuguese, and Mexican, were excluded.

### 3.1 Inclusion and exclusion criteria

Peer-reviewed articles published between the years 2000 and 2024 were considered and used in this study. Articles related to other assisted reproductive technologies were excluded. Articles addressing sex-sorted semen in cattle, sheep, and goats were included in this review. Furthermore, duplicates and studies addressing sex-sorted semen in other species, such as pigs, were also excluded from the data set.

## 4 Extinction status in South African livestock

South Africa is endowed with numerous indigenous breeds and is capable of developing local breeds to tackle meat demand under harsh environmental conditions (7). South African indigenous goat breeds include Nguni, SA veld, Tankwa, and Imbuzi (9). According to the DAD-IS database (9), these breeds are at risk of extinction and thus require immediate conservation intervention (see Table 1). The Nguni goat originated from the small East African indigenous goats and has a lop ear. Tankwa goats are wild animals that were discovered in the Tankwa Karoo National Park in South Africa's Northern Cape and are said to be well suited to their tough habitat (19), hence Tankwa is said to be the place of no water. The origin of SA goat veld is not yet known (9).

**Table 1:** The extinction status of South African indigenous livestock animals (9).

**Breed**	**Use**	**Origin**	**Local risk status**	**Conservation program**
**Indigenous goats' breeds**				
Bantu	-	Northern Transvaal, South Africa	Unknown	None
Edelziege	-	-	Unknown	-
South African goat	-	Limpopo	At risk	Mara research station
Kalahari red	Meat	-	At risk	None
SA veld goat	-	-	Unknown	None
Saffer	-	-	Unknown	None
Tankwa	-	Northern cape	Unknown	None
**Indigenous sheep breeds**				
Zulu sheep	Meat	KwaZulu Natal	At risk	Dundee and Makhathini
BaPed sheep	Meat	Limpopo	At risk	Mara
Namaqua Afrikaner	Meat	Northern cape	At risk	-
**Indigenous cattle breeds**				
Afrigus	Meat	-	Endangered	-
Afrikaner	Meat	-	Not at risk	-
Bonsmara	Meat	-	Not at risk	-
Bovelder	Meat	-	Unknown	-
SA ecotype breeds	-	-	Unknown	-

South African indigenous sheep have been used to develop locally developed breeds with the purpose of tackling adaptation challenges faced by exotic breeds (10). For instance, Ronderib Afrikaner has been used to develop Afrino and Van Rooy (9), while Damara was used to develop Meatmaster, and Bapedi was used to develop Bosvelder (20). Nevertheless, these breeds have been reported to face extinction due to uncontrolled crossbreeding taking place in rural farms where indigenous breeds are mostly found (21).

South African indigenous cattle include Afrigus, Afrikaner, Bonsmara, Bovelder, Shangaan, and Venda breeds (9). Afrigus is a locally developed breed that was bred by crossing Afrikaner and Angus (22). Afrikaner, on the other hand, is an indigenous breed in South Africa that was first registered in ~1907 in Ermelo and the Transvaal Department of Agriculture (23). Bonsmara, on the other hand, originated from South Africa and was particularly bred for economic production under an extensive system (24). Bovelder is a locally developed breed made to survive under harsh environmental conditions. This breed was developed by crossing Bonsmara and Afrikaner and Simmentaler and Sussex genetics (20). There are many ecotype breeds in South Africa including Zulu, Pedi, Shangaan, and Venda cattle breeds (25). These South African ecotypes were named after the tribe who were keeping these breeds.

## 5 An overview of sexed semen

Pre-selection of semen based on the sex chromosomes (X- and/or Y-bearing chromosomes) is of paramount importance to obtain the desired sex of the offspring and avoid animal wastage as much as possible (26, 27). The issue of animal wastage is the pivotal factor that needs to be avoided particularly when the breed is endangered (11).

The cattle industry has benefited from sperm sexing technology (28). For instance, dairy farmers are currently able to pre-select the desired sex to increase replacement heifers and avoid bulls because bulls have a low economic value in dairy farms (29). Therefore, sperm sexing is a vital tool that will have a positive impact on the livestock industry and conservation programs (27). However, this method is associated with numerous flaws, such as the survival of the sperm cells, in comparison to the unsexed sorted sperm (30).

The sperm sexing technique relies on the differences between the nuclear constituency of X and Y chromosomes bearing spermatozoa (14). The benefits of using sex-sorted semen to conserve endangered breeds include improved semen quality after sex-sorted sperm using flow cytometry, genetic progress following the use of sex-sorted semen, increased females for breeding, increased herd and number of bulls to create revenue, and their use for seedstock purposes (27).

There are numerous techniques developed to date to sex livestock semen. These techniques include albumin gradient/gradient swim-down procedure, Percoll density gradient method, swim-up procedure, free flow electrophoresis, the identification of H–Y antigen, sperm sorting based on the volumetric differences, centrifugal counter current distribution, immunological approaches, proteomics approaches, and flow cytometry (31). Gaur et al. (31) described the swim-up procedure, and the albumin gradient method uses the size and motility of Y-bearing sperm cells to sex sperm cells, while the Percoll density method uses the sedimentation density of X-bearing sperm cells. The disadvantage of the albumin gradient method is that it can only separate human sperm cells. Free flow electrophoresis makes use of electric charges where X-bearing sperm cells are negatively charged, and the identification of H–Y antigen uses proteins that are available in the X- or Y-bearing sperm cells' surface. Flow cytometry is the only commercialized method of sexing semen and uses LASER to stimulate fluorescent dye that fixes to the DNA in spermatozoa. The disadvantage of flow cytometry is that it is associated with high sperm damages.

### 5.1 Flow cytometry

**Figure 2:**
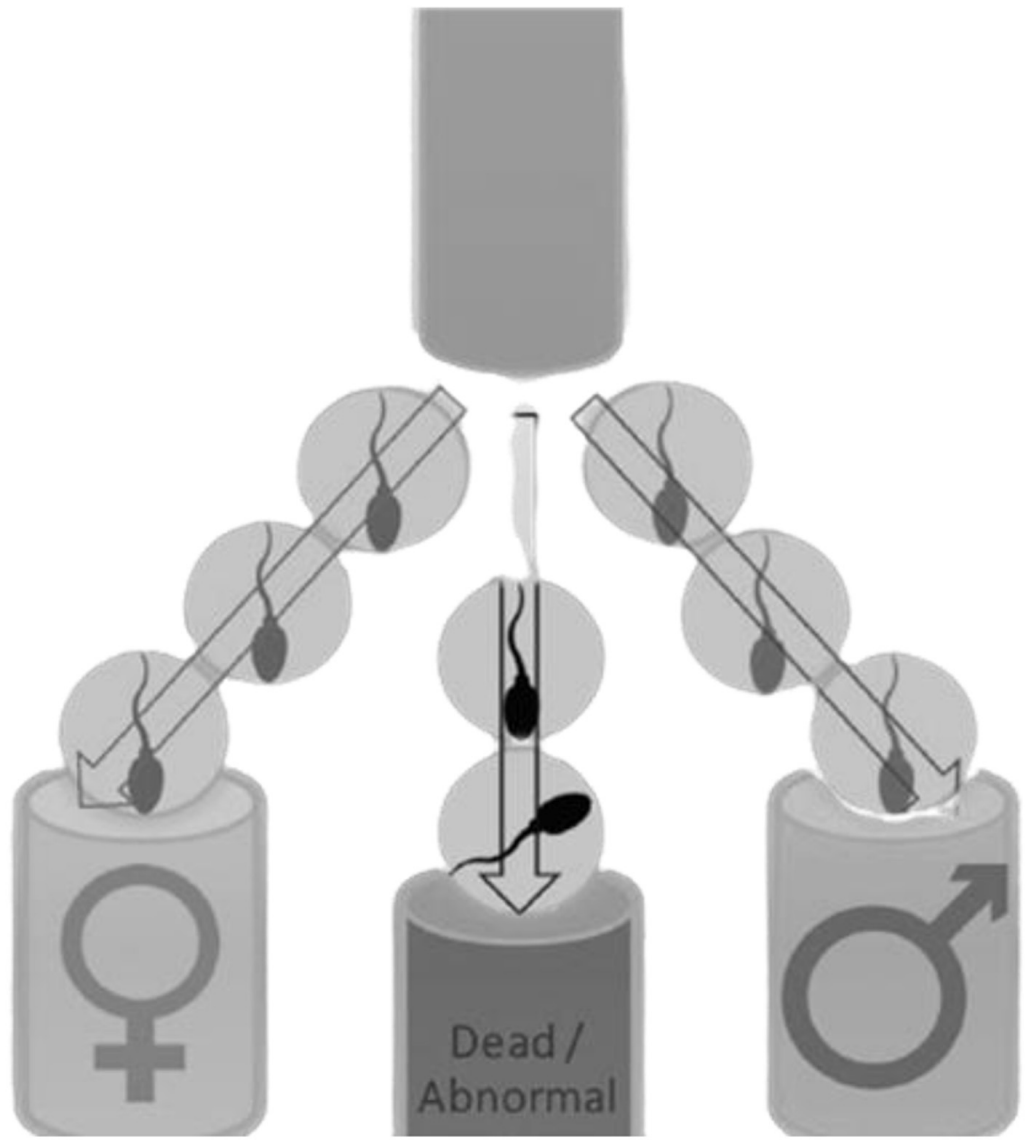
Sperm sex sorting process. Reproduced from Hofmeyr (2021); with permission from Izak Hofmeyr, Stockfarm Magazine and Dr Fanie Steyn, Ramsem SA (34).

There is a 50% probability of producing either male or female offspring in mammalian animals because half of the ejaculate contains either Y- or X-bearing spermatozoa (32). Males are known to produce two types of sperm cells (X or Y); in which, when the Y-bearing sperm fertilizes an egg, it results in the birth of a male. Whereas, when the X-bearing sperm cell fertilizes an egg, it will result in the birth of a female (31). Early research found that ram spermatozoa (X or Y) has some differences, making it easier to predetermine gender. Among the few differences, the female (X) sperm contains higher DNA (4.2%) content when compared to the Y-bearing spermatozoa (23). Moreover, the size of X-bearing sperm cells is larger than Y-bearing sperm cells, while Y-bearing sperm cells have higher motility than the X-bearing sperm cells (33). These differences make it easier to separate X- and Y-bearing sperm cells (see Figure 2). Therefore, predetermining the gender of the offspring can assist in speeding up the conservation of domestic animals through desired offspring and avoiding animal wastage (35). Flow cytometry is the only proven method to acquire +90% gender accuracy (32). However, this method (flow cytometry) is harmful to sperm cells, particularly influencing sperm cell viability (27).

## 6 Prospects of sex-sorted semen in conservation programs

Semen analysis is a vital aspect in the evaluation of semen used in assisted reproductive technology such as sex sorting (36). Diverse factors can affect the quality of semen in different animal species, not to mention sex-sorted semen. However, to allow faster genetic progress on endangered animals, it is important to generate productive animals while reducing wastage through sex-sorted semen (35). Sperm sex sorting is the only technology that is capable of distinguishing X- and Y-bearing chromosome sperm based on DNA content differences. The application of sex-sorted semen can accelerate genetic progress in all species of agriculture but lower animal waste and allow farmers to choose how to best increase the economic features of their herds (11), hence some researchers consider it a game changer (34).

Sex sorting sperm processes select a physiologically better collection of sperm from ejaculate for both *in vitro* and *in vivo* objectives, resulting in superior sperm fertilizing capacity after sorting ram sperm cells (37). During the attempt to conserve domestic species, pregnancy and parturition remain vital to increasing the population. The pregnancy outcome is critical at this stage and can result in animal wastage because of the wrong sex of the offspring. There is a fixed probability of 51:49 in the sex ratio favoring the male gender in domestic livestock animals. This genetic trait cannot be manipulated effectively through natural or artificial insemination (38). Therefore, it is important to produce offspring that will effectively contribute to the conservation program, which can be found in Table 2.

**Table 2:** Kidding rates, lambing rate, calving rate, and sex accuracy after artificial insemination with sexed semen.

**Species**	**Sperm type**	**Number of females inseminated**	**Kidding/ lambing/ calving rate (%)**	**Sex/gender accuracy (M:F)**	**Effects of sperm sexing**	**Reference**
Goats	X-bearing	8	1 (12, 5)	0:1	Accurate, with a low conception rate	(39)
	Y-enriched	5	4 (1 × twin; 80)	4:1		
Sheep	X-bearing	139	-	93% females	Sperm sexing was accurate	(40)
	Y-enriched	140	-	97% males		(40)
	X-bearing		74.15	91.90	Sperm sexing was accurate	(41)
Cattle	X-bearing	-	30 (66.7)	Female: 21 (70.0) Male: 9 (30.0)	Accurate	(42)
	X-bearing	-	58	47/58 (81.03)	Accurate	(43)
	Y-enriched	-	51	37/51 (72.54)	Accurate	(43)
	X-bearing	35	45.8	100	Accurate	(44)
	Y-enriched	18	49.1	62.5	Not accurate	(44)

The above table concludes that sex-sorted semen is accurate in terms of gender pre-selection resulting in more than 97% accuracy in some studies (40). Nevertheless, the low pregnancy rate is still problematic and needs more clarity through research studies.

### 6.1 Semen quality following sex sorting using flow cytometry and preservation

Semen quality after sex sorting using flow cytometry is well known (45). Flow cytometry selects male and female sperm cells and discards dead and abnormal sperm cells during the process (34). This process leads to the hypothesis that the sex-sorted semen has lower sperm quality in comparison to the non-sex-sorted semen (46), which is because the speed, pressure, laser light, electrical charging, processes involved in sorting semen deviation, and changes in the medium jointly lead to defects that can damage sperm cells (31, 47).

It is noteworthy that, fewer (if any) studies evaluated sperm quality following sex sorting semen and liquid preserved. Liquid-preserving semen is known to yield better results when compared to frozen-thawed semen (Table 3) (50). This technique has been used in the artificial insemination program in many research stations and farms near the research stations and has proven to yield better results (53). Therefore, the same application following sex sorting semen can be useful to evaluate if better results cannot be obtained.

**Table 3:** Sperm quality following sex sorting using flow cytometry and analyzed with computer-aided sperm analysis.

**Species**	**Sperm parameters**	**TM (%)**	**VAP (μms−1)**	**VSL (μms−1)**	**VCL (μms−1)**	**STR (%)**	**LIN (%)**	**References**
Goats	Fresh sexed	86.3	113.3	87.9	132.8	77.3	65.8	(45)
	Chilled unsexed	64.3	113.6	73.6	132.0	66.8	59.4	(48)
	Chilled sexed	-	-	-	-	-	-	-
	Cryopreserved, unsexed	67.80	86.08	67.78	140	77.40	51.00	(49)
	Cryopreserved, sexed	41.6	54.4	44.7	69.1	80.9	62.0	(45)
Sheep	Fresh unsexed	91.30	79.57	52.50	137.78	62.53	57.14	(50)
	Chilled unsexed	85.15	47.94	27.39	89.86	55.7	35.77	(51)
	Chilled sexed	-	-	-	-	-	-	-
	Cryopreserved, unsexed	46.97	64.39	-	-	-	57.23	(52)
	Cryopreserved, sexed	-	-	-	-	-	-	-
Cattle	Fresh sexed	-	-	-	-	-	-	-
	Chilled unsexed	-	-	-	-	-	-	-
	Cryopreserved, unsexed	-	26.30	22.02	43.11	77.74	40.12	(46)
	Cryopreserved, sexed	-	25.97	20.97	42.75	70.98	32.15	
Effect on sexed sperm parameters		±50% decrease of TM	±50% decrease of VAP	±50% decrease of VSL	±50% decrease of VCL	No much differences	No much differences	-

Critical sperm parameters (TM, VAP, VSL, and VCL) could be affected by sex-sorting processes. These damages cannot be reversed since they involve plasma membrane proteome and mitochondrial protein changes (54). Sperm motility is the total number of sperm motile and is considered essential for fertility prediction (55). Sperm motility has been found to be sensitive and can be affected by *in vitro* sperm handling (56), and it is shown in Table 3 following sexing semen. The velocity parameters, on the other hand, are critical for the fertilizing ability of the sperm cell (57).

## 7 Limitations associated with sexed semen

Sexing semen requires a sophisticated machine along with high maintenance costs (31). Furthermore, the quality of frozen-thawed sheep and goats' spermatozoa is very poor, forcing researchers and farmers to prefer sexing fresh semen, which requires stations to be in closer proximity to the farms. It also appears that this technology requires a high selection intensity for good quality traits, hence purchasing sex-sorted semen from the nearby station can increase the chances of genetic base and inbreeding (58). Therefore, this review also examines the limitations of sexing semen and focuses on fertility outcomes after the utilization of sex-sorted semen and different artificial insemination techniques, pregnancy and embryonic loss following the use of sex-sorted semen, the proteomic shift in sexed semen and its influence, impaired sperm protection, and the decline in the antioxidant level (total superoxide dismutase (SOD), catalase (CAT), glutathione peroxidase (GSH-Px), and glutathione reductase) in the semen following sexing semen.

### 7.1 Fertility outcomes after using sex-sorted semen and different artificial insemination techniques

Appropriate sperm dose for the type of insemination (laparoscopic or transcervical AI) and synchronization protocols are needed that would provide comparable results to conventional semen when using sex-sorted semen (35). There are factors reported to influence the pregnancy rate following sexing semen such as the number of doses. A study by González-Marín et al. (35) found that increasing the number of doses can improve the calving rate. However, more studies are still required to prove such results.

Based on Table 4 below, conception and the lambing rate are still not satisfactory despite the method of artificial insemination used. Despite numerous efforts made to use sexed semen in ruminants, there is still a lack of information on ovine, particularly cryopreserved and sexed semen. Studies in sheep have reported on the conception rate following fresh sexed semen, cryopreserved sexed semen, and chilled sexed semen. However, the conception rate remains below expectation levels. Similar problems have been reported when ram or buck semen is cryopreserved (56). A molecular study by Peris-Frau et al. (60) observed a loss of proteins, lipids, and ions during cryopreservation and speculated that as a primary cause of poor post-thawed sperm quality. Therefore, poor conception rate in sheep and goats might be due to cryopreservation and sexing semen.

**Table 4:** Lambing rates following artificial insemination in sheep and goats using frozen-thawed sex-sorted semen.

**Species**	**Storage type**	**Artificial insemination**	**Conception rate (%)**	**Lambing /calving rate (%)**	**Effects of AI method sex-sorted semen fertility**	**Reference**
Goats	Fresh sexed	Deep cervical	74.15	74.15	High lambing rate	(41)
	Cryopreserved sexed	-	-	-	-	-
Sheep	Cryopreserved sexed	Laparoscopic	-	35.8	Low lambing rate	(35)
	Cryopreserved sexed	Intrauterine laparoscopy	39	35	Low lambing rate	(37)
	Cryopreserved sexed	Laparoscopic	-	32.8	Low lambing rate	(35)
	Cryopreserved sexed	Cervical insemination	13.5	-	Low conception rate	(59)
Cattle	Cryopreserved sexed	Timed artificial insemination	78.4 (29/37)	-	Low calving rates	(12)
	Cryopreserved non-sexed	Timed artificial insemination	(62.9) 22/35	-	Low calving rate	(12)

An improvement has been observed in cattle both in terms of the use of sexed semen and acceptable conception rate especially in dairy cattle (12). This improvement might be due to good sperm quality following freezing when compared to other ruminants (61).

### 7.2 Pregnancy and embryonic loss following the use of sex-sorted semen

It has been observed that a lesser number of sperms per insemination using sexed sperm lowers fertility, which may result in the loss of embryos or pregnancy *in vivo* (62). It is critical to identify whether the loss of pregnancy in cows is due to significant modifications in sperm function caused by the procedures employed for preparing frozen-thawed sperm for sorting, the sorting process itself, and re-freezing, or an amalgamation of these factors. However, plausible explanations for the poor pregnancy rates following AI using frozen-sexed re-frozen sperm include decreased sperm function, incorrect AI scheduling in relation to ovulation, or an insufficient dosage of viable sperm. Underwood et al. (63) hypothesized that the addition of two freezing procedures to sorting may cause further changes to the sperm cell, compromising their ability to fertilize and, in cases where fertilization is successful, leading to embryos with a lower ability to sustain a pregnancy. Another theory is that, in addition to environmental effects or maybe poor bull selection, pregnancy loss may be attributed to diminished sperm function induced by sex sorting and re-freezing, resulting in low embryo quality. Furthermore, Magopa et al. (12) identified that another factor associated with pregnancy loss in cows could be the use of sexed sperm with sperms that sustained injury during the sorting procedure, which could have resulted in a further reduction in sperm viability in the cow's reproductive tract. Pohler et al. (64) argued that the mechanisms causing pregnancy loss in cows might relate to embryo development or a lack of additional embryonic membrane formation since this happens around the time of embryo attachment and the onset of placentation.

A similar pattern of embryo losses when implementing sex-sorted sperm has been observed in *in vitro* embryo production facilities (see Table 5), which are characterized by liaised embryos at the cleavage, morulae, or blastocyst stages. Similar to *in vivo* cases, embryo loss *in vitro* may be related to the quality of sperm used for fertilization, the effect of the sorting procedure and its environment, the quality of the oocyte and its source, the *in vitro* environment, and the media used for *in vitro* embryo production, particularly at the *in vitro* maturation (IVM) level, keeping in mind that they are also supplemented with certain additives such as bovine serum albumin and fetal bovine serum. According to Sithole et al. (67), bovine serum albumin has a low lipid concentration, but fetal bovine serum has a high lipid content, which results in greater lipid accumulation in oocytes. This finding induces alterations in mitochondrial and lipid dynamics at the IVM level, which may have a deleterious impact on oocyte development rates and embryo lipid buildup. When it comes to embryo development, the influence of media supplements cannot be completely prevented. The components of maturation medium and culture conditions can influence or even regulate the meiotic regulation of oocytes in mammals (67). The results in embryo loss development may also be tempered by the fact that most laboratory setups utilize denuded presumptive zygotes with a vortexing mechanism after fertilization (67), of which the removal of cumulus cells using this mechanism damages the cytoplasm.

**Table 5:** The effect of sex-sorted semen on *in vitro* embryo production and *in vivo* reproductive performance.

***In vitro*** **reproductive performance**					
**Species**	**Semen Preservation method**	**IVC rates (%)**	**Total blastocyst (%)**	**Embryo loss (%)**	**References**
Goats	-	-	-	-	-
Sheep	-	-	-	-	-
Cattle	Cryopreserved non-sexed	735 (69.0)	319 (30.0)	416 (43.4)	(65)
	Cryopreserved sexed	793 (70.6)	250 (22.3)	543 (68.5)	(65)
	Cryopreserved sexed	746/1025 (73)	281/1025 (27)	465/746 (62)	(43)
	Cryopreserved sexed	1025/1577 (65.0)	125/1025 (12.2)	900/1025 (88.0)	(66)
***In vivo*** **reproductive performance**					
**Species**	**Type of semen**	**Conception rate (%)**	**Lambing/calving rate (%)**	**Pregnancy loss (%)**	**References**
Goats	Cryopreserved unsexed	74.15 (66/89)	-	-	(41)
	Cryopreserved sexed	65.78 (25/38)	-	-	
Sheep	Cryopreserved unsexed	-	-	-	-
	Cryopreserved sexed	-	-	-	-
Cattle	Cryopreserved non-sexed	22/35 (62.9)	-	5/22 (22.7)	(12)
	Cryopreserved sexed	33/45 (73.3)	30/45 (66.7)	9	(42)
	Cryopreserved sexed	29/37 (78.4)	-	8/29 (27.6)	(12)

Minimal research has been done in sheep and goats to investigate *in vitro* fertility following sexing semen. Vianna et al. (68) reported fewer *in vitro* embryo production studies in both sheep and goats in Africa. Therefore, more *in vitro* embryo production studies are still needed in sheep and goats and the use of sexed semen thereafter. This finding will assist not only in improving breeds but also in conserving endangered breeds.

### 7.3 The proteomic shift in sexed semen and its influence

Sexing semen does not only reduce sperm motility parameters but also shifts proteome content, particularly after cryopreservation. For instance, Mostek et al. (54) concluded that sexing semen may result in glycolysis, OXPHOS, and the maintenance of adenylate energy charge. Furthermore, sexed semen in rams has been found to yield fewer mitochondrial proteins (69). It is well known that mitochondria in a sperm cell are responsible for the energy, hence its damage can lead to sperm death. It also appears that mitochondria are the main intracellular source of ROS in the form of superoxide anions through the electron transport chain; however, such ROS generation takes place in a regulated way (70). Moreover, proteins involved in sperm capacitation, acrosome reaction, and sperm fusion and the reduction of sperm surface protein have been observed (54). This finding may lead to lesser sperm protection, impaired gamete recognition, and disrupted cell signaling. When it comes to fertility, although there is limited information, Kasimanickam et al. (71) observed significantly higher sperm proteomes in high-fertility bulls when compared to infertility bulls, which means that the proteome shift in sexed semen might influence the fertility of the sperm cells. This observation is critical because sperm proteins are essential for sperm-egg fusion and embryonic development (71). For instance, greater generation of ROS is driven by the electron leakage from the mitochondria electron transport chain with a consequent decrease of molecular oxygen to form the superoxide anion (72).

#### 7.3.1 Impaired sperm protection

The sperm membrane is the first sperm organ influenced by any artificial handling of semen (56). Therefore, it is very critical to ensure sperm protection during semen handling. In cases of cryopreservation or liquid storage, this protection has been achieved through milk or egg yolk inclusion in the extenders. Nevertheless, it has been reported that sexing semen also leads to impaired sperm protection (54). It is still not clear whether egg yolk or milk can protect sperm cells during various sexing processes.

### 7.4 A decline in the antioxidant level (total superoxide dismutase, catalase, glutathione peroxidase, and glutathione reductase) in the semen following sexing semen

Semen consists of natural enzymatic antioxidant defense system, such as catalase, superoxide dismutase, reduced glutathione (GSH), and glutathione peroxidase, and non-enzymatic system such as vitamin C, cysteine, and glutathione. Their function is to protect ejaculated spermatozoa from the notorious effects of ROS (73). Reactive oxygen species (ROS) refers to any molecule capable of oxidizing biological substrates comprising fats, protein, and DNA and can be in the form of radicals characterized by unpaired valency electrons such as superoxide anion, hydrogen peroxide, and or peroxynitrite (74). These are metabolites of oxygen and include superoxide anion, hydrogen peroxide, hydroxyl and hydroperoxyl radicals, and nitric oxide (75). The main ROS produced in the semen includes hydrogen peroxide (H2O2), superoxide anion (O^·^), and hydroxyl radical (OH) (76). Cryopreservation also alters the distribution of proteins, such as glutathione peroxidase, glutathione reductase, and superoxide dismutase, that are necessary to scavenge ROS (60). A recent study by Guo et al. (46) observed a massive reduction of natural antioxidants in sexed semen (Table 6). This finding implies that poor conception rate and embryo development following the use of sexed semen can be caused by lower antioxidant defense against ROS. Nevertheless, there is still a need for more studies evaluating the antioxidant capacity following sexing semen particularly in sheep and goats.

**Table 6:** Semen antioxidant capacity following semen sexing.

**Species**	**Type of semen**	**Catalase CAT (U/mL)**	**Superoxide Dismutase (SOD (U/mL))**	**Glutathione [GSH (U/L)]**	**Plasma Glutathione Peroxidase [GSH-Px (U/L)]**	**Oxyrase**	**Humanin**	**Reference**
Goats	Unsexed	-	-	-	-			-
	sexed	-	-	-	-			-
Sheep	Unsexed	-	-	-	-			-
	sexed	-	-	-	-			-
Cattle	Unsexed	3.79	1.66	55.51	121.2	639.50		(46), (77)
	sexed	0.73	0.45	23.78	4.33	-		

*In vitro* sperm handling, such as cryopreservation and sperm sexing, leads to the development of free radicals as a result of excess ambient air (78). Oxyrase enzyme is derived from the cytoplasm membrane of E. coli that is known to produce anaerobic conditions. This enzyme has been used to protect the motility of frozen-thawed sperm cells in bulls and is reported to sustain total antioxidant capacity and total motility (77). However, this *E. coli*-derived enzyme has not been tested in sexed sorted semen. Humanin, on the other hand, protects sperm against oxidative stress and apoptosis (79). Supplementing Humanin in buffalo's sperm cells has been found to improve freezability despite buffalo sperm sensitivity to cryopreservation (79). Therefore, these enzymes have the potential to improve sex-sorted sperm quality; however, further studies need to be conducted.

## 8 Different ways to improve sex-sorted semen quality and fertility

Despite numerous limitations of sex-sorted semen, there are alternatives that can be harnessed to improve sex-sorted semen. This review will address the application of antioxidants both in the extender and feed supplementation to improve sex-sorted semen and the application of nanotechnology to improve sexed semen.

### 8.1 The application of antioxidants both in the extender and feed supplementation to improve sex-sorted semen

Sperm sexing is a good ART; however, it is associated with the production of oxidative stress (OS) and DNA fragmentation (SDF) (14, 80). According to Pintus et al. (81), oxidative stress occurs in the sperm cell when the amount of reactive oxygen species overcomes the level of antioxidants. Guo et al. (46) observed a relatively low antioxidant enzyme (GSH; SOD; CAT; and GSH-Px) and the rate of fertilization activities in the sex-sorted semen. In this study, it was concluded that these less enzymatic activities observed in the sex-sorted semen were caused by the complex processes of sorting semen.

Numerous studies have attempted to scavenge reactive oxygen species in semen through supplementing antioxidants either by feed supplementation (82) or through extender supplementation (83) with noticeable improvements (see Table 7). There are many anti-oxidant sources that have been used to scavenge reactive oxygen species when however, until now, no conclusion can be drawn whether the natural antioxidant can improve sex-sorted semen or not. Sex sorted semen will assist the pioneer sex-sorting semen method to play a possible role in the conservation of endangered species.

**Table 7:** An influence of antioxidants following sex sorting semen.

**Species**	**Antioxidants**	**Preservation method**	**Effects**	**References**
Goats	Ascorbic acid glucoside, glutathione, and vitamin C	Cryopreservation	Improved lambing rate	(39)
Sheep	-	-	-	-
	Linoleic Oleic	Cryopreservation	No differences (*p* > 0.05) between the treated group and the control (no-sexed)	(37)
Cattle	Vitamin C (VC) and lycopene (Lyc)	Liquid preservation	Improved the fertilization capacity of sex-sorted sperm during the IVF procedure.	(76)

### 8.2 The application of nanotechnology to improve sexed semen

Nanoparticles can be defined as particles shaped in very small sizes with flexible fabrication and a high surface area ratio (84). Nanoparticles can be made from numerous materials including metals, polysaccharides, and proteins and have gained an interest in medicine. Furthermore, nanotechnology has been marked as a promising tool for improving assisted reproductive technologies (85). Assisted reproductive technologies that have gained more attention recently include artificial insemination, *in vitro* fertilization, *in vitro* embryo production, *in vitro* culture of follicles, and semen sex sorting/pre-gender selection (86). Pre-gender selection has numerous advantages reported, including reducing genetic wastage, breeding desired gender, and improving genetic progress either for sales or for replacement heifers (27).

Preselecting gender presents a promising approach in determining gender before artificial insemination (87). However, despite interest in sexed-sorted semen in the research industry, conception rates remain below expectations (27). Irreversible damages that occur to the sperm cells as a result of sexing semen might explain the low conception rate following the use of sex-sorted semen (59). This finding led to more research looking to improve sex-sorted semen either as fresh, chilled, or frozen thawed (37, 39). Several studies in different species have reported fertility improvement when nanoparticles are used to improve semen (88, 89). For example, in pigs, there was an improvement when nanoselection was used during semen sex selection by removing unwanted abnormal sperm cells and hence improving fertility (90).

## 9 Discussion

This review aimed to explore the prospects of X- and Y-sexed semen in ruminant livestock and to investigate its potential to conserve threatened ruminants. This review confirmed that there are many South African indigenous ruminants (cattle, sheep, and goats) that are critically endangered. It is noteworthy that there are conservation programs in place to curb this problem. Ngcobo et al. (10) reported that some of these programs are ineffective. Sexed semen has the potential to speed conservation programs although numerous inputs to improve sexed semen quality are still required. For instance, the commercially employed method (flow cytometry) for sorting semen kills the sperm cells during selection (27). Other studies have reported a noticeable decrease in sperm quality following sexing semen (45, 46).

Despite the low conception rate following the use of sexed semen in goats, sheep, and cattle, the method is accurate (39, 40, 44). This finding, therefore, necessitates more innovation studies to come up with a sexing technology that will protect sperm cell injuries during sorting, ehich was supported by the evidence from Silva et al.'s (45) study in frozen-thawed goats' sperm cells and Guo et al.'s (46) study in frozen-thawed cattle's sperm cells. However, the type of insemination used in goats, sheep, and cattle also affects the conception rate following the use of sexed semen (12, 35, 37, 41, 59).

Sperm quality after conception or during embryo production is vital to avoid embryo loss (91). For instance, Wilson et al. (66), reported embryo loss of ~88% when frozen-thawed sexed semen was used in cattle. The recent study by Bermudez et al. (91) reported a significant difference when fresh-sexed semen and frozen-thawed sexed semen were used with no effect of parity. The decline in the antioxidant level (total superoxide dismutase, catalase, glutathione peroxidase, and glutathione) in the sexed semen might be a primary cause of poor sperm quality and the failure to maintain embryonic growth. Moreover, adding ascorbic acid, glutathione, linoleic acid, and lycopene could be able to protect sperm cells during the sexing method (37, 39, 92).

The role of sorted sperm has been observed in the dairy sector more than in other livestock sectors such as sheep, goats, and beef cattle (93). The use of sex-sorted semen in the dairy sector has been driven by the demand to produce replacement heifers instead of bulls since dairy bulls are expensive to keep. In sheep and goats, this technology has not been used intensively. This finding might be due to poor frozen-thawed semen in sheep and goats and also poor conception rate following artificial insemination (94). However, this method can play a substantive role in sheep and goats, particularly in their conservation programs and to improve replacement ewes and the production of males in feedlot set-ups.

## 10 Conclusion

Studies investigating both fresh and cryopreserved sexed semen in sheep and goats are still lacking in comparison to that of cattle. However, this technique can play a significant role in the conservation programs of endangered ruminant livestock. For instance, based on the literature, sexing semen before artificial insemination can improve ruminants' livestock genetic material and play a significant role in conserving and reducing genetic wastage. Nevertheless, there are limitations associated with sexing semen, such as low semen quality and conception rate following sexing semen, opening room for further studies. Enormous research studies are only focusing on the flow cytometry for sperm sexing with lesser sperm motility and quality following sex sorting. On the other hand, flow cytometry is considered a commercial method of sexing semen, however, with a huge loss of sperm motility and velocity parameters. Moreover, this method (flow cytometry) is associated with high purchase and maintenance costs. Therefore, there is a need to develop and commercialize an alternative method for sperm sexing that will result in comparable sperm motility recovery to the fresh semen. The use of antioxidants to protect and improve sex-sorted semen can be beneficiary and have the capacity to improve the conception rate following the use of sex-sorted semen.

## Data availability statement

The original contributions presented in the study are included in the article/supplementary material, further inquiries can be directed to the corresponding author.

## Author contributions

JN: Conceptualization, Methodology, Writing – original draft. TN: Writing – review & editing. SS: Writing – review & editing, Methodology. BM: Writing – review & editing. TM: Writing – review & editing. FR: Writing – review & editing. TC: Writing – review & editing. KN: Writing – review & editing.
